# The Cooccurrence of Obesity, Osteoporosis, and Sarcopenia in the Ovariectomized Rat: A Study for Modeling Osteosarcopenic Obesity in Rodents

**DOI:** 10.1155/2017/1454103

**Published:** 2017-06-01

**Authors:** Zahra Ezzat-Zadeh, Jeong-Su Kim, P. Bryant Chase, Bahram H. Arjmandi

**Affiliations:** ^1^Department of Nutrition, Food, and Exercise Sciences, Florida State University, Tallahassee, FL, USA; ^2^Center for Advancing Exercise and Nutrition Research on Aging (CAENRA), College of Human Sciences, Florida State University, Tallahassee, FL 32306, USA; ^3^Department of Biological Science, Florida State University, Tallahassee, FL, USA

## Abstract

**Background:**

Obesity, osteoporosis, and sarcopenia may individually occur due to age-related gradual alterations in body composition. This study investigates the cooccurrence of these age-related diseases in female animals with low levels of ovarian hormone in the absence of complex multifactorial process of chronological aging.

**Methods:**

Thirty-six 5- and 10-month-old female rats were chosen to model pre- and postmenopausal women, respectively. Rats were divided into three treatment groups in each age category—sham, ovariectomized (ovx), and ovx + E_2_ (17*β*-estradiol, 10 *μ*g/kg)—and were pair-fed. Volunteer wheel running activity, body composition, bone microstructure, serum C-telopeptides of type I collagen, bone specific alkaline phosphatase, E_2_, and gastrocnemius and soleus muscles were analyzed.

**Results:**

The cooccurrence of osteoporosis, sarcopenia, and obesity was observed in the older ovx rats associated with a significant (*p* < 0.05) increased fat mass (30%), bone loss (9.6%), decreased normalized muscle mass-to-body-weight ratio (10.5%), and a significant decrease in physical activity (57%). The ratio of tibial bone mineral density to combined muscle mass was significantly decreased in both ovx age categories.

**Conclusion:**

Ovariectomized rat could be used as an experimental model to examine the effect of loss of ovarian hormones, while controlling for energy intake and expenditure, to conduct obesity and body composition translational research in females without the confounding effect of genetic background.

## 1. Introduction

Obesity, osteoporosis, and sarcopenia are three disease conditions that may occur due to age-related gradual alterations in body composition [1, 2]. Excess fat mass has been suggested as a risk factor for the gradual decline in muscle mass and the combination of both conditions has termed sarcopenic obesity [1]. Interestingly, in spite of the traditional belief that obesity was protective against osteoporosis, a more recent, growing body of evidence indicates that fat mass is negatively correlated with bone mass, suggesting that excess body fat has deleterious effects on bone [3, 4].

In women and certain animal models such as rat, estrogen is considered a major regulator of adipose tissue development and its deposition [5–9]. In addition to the direct role of estrogen on adipose tissue [6–8], it may also indirectly affect adiposity via regulation of food intake and energy expenditure by affecting hypothalamus functions [5, 9]. Moreover, ovarian hormone deficiency often not only is associated with an increase in fat mass, but also is linked to an accelerated loss of bone mass and development of osteoporosis [2, 3].

While the effect of estrogen on bone is better understood, its effect on other tissues including skeletal muscle is poorly understood and needs further investigation. Available findings suggest both direct and indirect roles for estrogen in muscle protein synthesis or maintaining lean tissues in both human and rodents [10, 11]. However, there is disagreement in available literature related to the effects of estrogen on skeletal muscle mass. Declined, increased, and maintained skeletal muscle mass has been reported following ovarian hormone deficiency and administration of estrogen, making it difficult to interpret the role of estrogen in the progressive decline of muscle mass which is clinically known as sarcopenia [12–15]. Differences in energy intake and expenditure—two main factors that affect body weight and that vary as a consequence of ovarian hormone deficiency—may partially explain discrepancies among previous findings. Additionally, the effects of ovarian hormone may be age specific and impact the role of estrogen manipulation on lean mass in growing versus mature animals [13, 14]. The duration of the estrogen deficiency [16] and specific metabolic alterations following ovariectomy in mice and rats could also be reasons of these inconstancies in the available literature [17]. It is likely that ovarian hormone deficiency results in an increased adiposity in the face of bone and muscle loss due to the adverse effect on osteoblastogenesis and myogenesis [11, 18]. These unfavorable changes in body composition may interact negatively to predispose women with low levels of ovarian hormone to impaired physical function and age-related chronic disease.

The cooccurrence of obesity, osteoporosis, and sarcopenia—three diseases that occur with advancing age and which may be affected by fluctuations in ovarian hormone production or its deficiency—has not been investigated in females while controlling for energy intake and expenditure. This study addresses two important questions: (1) do these three age-related disease conditions cooccur in females with low levels of ovarian hormone? (2) How the presence or absence of estradiol (E_2_), the most biologically active form of estrogen, affects muscle, bone, and fat mass in an experimental model without the confounding effects of chronological aging or genetic background?

To investigate these issues, we used Sprague-Dawley (SD) rats that were 5 or 10 months old at the beginning of the study to model pre- and postmenopausal women, respectively. Using these two age groups, we were able to compare the impact of estrogen deficiency along with advancing age without any sign and complexity of chronological aging that negatively affects body composition. We hypothesized that ovarian hormone deficiency slows resting metabolic rate and energy expenditure resulting in an increased adiposity and a body weight (BW) gain while exerting catabolic effects on lean mass, including bone mass, in both young and old experimental categories. Furthermore, we postulated that this hypothesis was independent of age. To test this hypothesis, we controlled the effects of ovarian hormone deficiency on food intake by pair-feeding all of our ovariectomized (ovx) animals to the mean food intake of sham-operated rats. We also measured changes in physical activity in response to the interventions. The study was concluded once significant loss of whole body bone mineral density (BMD) was observed. We also measured bone mineral content (BMC) of whole body, tibia, and 4th lumbar vertebra and their microstructural properties. We examined sarcopenia by measuring lean and skeletal muscle mass and adjusting the values of skeletal muscle mass by weight as the most recommended diagnostic approach to determine presarcopenia, according to the European Working Group on Sarcopenia in Older People (EWGSOP). For our investigation we selected two hindlimb skeletal muscles: the soleus (SOL) that is mostly comprised of slow-twitch muscle fibers (type I) and the gastrocnemius (GAS) that is mostly comprised of fast-twitch muscle fibers (type II). The combined muscle mass of these two skeletal muscles that either are attached (SOL) or span the tibia (GAS) was used in computing the ratio of tibial BMC to muscle mass (MM). Although there are a number of studies that have evaluated the effects of ovarian hormone deficiency on body weight, fat, muscle, and bone status, to our knowledge this is the first study that addresses osteosarcopenic obesity in experimental model and evaluates the effects of the presence or absence of estradiol on these components of body composition, while simultaneously controlling the food intake and measuring physical activity in two different aged cohorts of rats.

## 2. Methods

### 2.1. Animals and Diet

Animal protocol was approved by the Institutional Animal Care and Use Committee at Florida State University. A total of thirty-six (*n* = 36) 5- and 10-month-old female SD rats were divided into sham, ovariectomized (ovx), and ovx + E_2_ (immediately after surgery 10 *μ*g/kg BW 17*β*-estradiol was injected subcutaneously twice per week) in each age group (*n* = 6 per treatment group). During the study period animals were pair-fed to the mean food intake of the sham with a semipurified control diet (AIN-93M) and weighed weekly until the occurrence of significant bone loss in ovx rats, which was after 3.5 months (age 8.5 months) for young and after 5 months (age 15 months) for older animals. Younger and older ovx + E_2_ groups received estradiol for the full duration of the study (3.5 months or 5 months, resp.). Rats were provided with free access to continuous voluntary running wheels in their individual cages for 24 hours, within 24 to 48 hours after E_2_ injection, one week before sacrifice. These running wheels were connected to a distance counter to measure animals' voluntary running activity.

### 2.2. Tissue Collection

At the termination of the study, animals were anesthetized with ketamine/xylazine (100 mg/5 mg/kg BW) and bled from their abdominal aortas. Tissues, including bone, muscle, liver, heart, and uterus, were harvested. An incision through the skin from the medial side of the thigh to the abdomen was made. The skin was then reflected to expose the muscles of the lower leg. An incision was made along the white facial line demarcating lateral aspects of the lower leg, from the ankle to 3–5 mm proximal to the ankle. GAS and SOL muscles from hindlimb were isolated from the distal end using forceps and scissors and weighted. Bone specimens including vertebrae and tibiae were collected, cleaned of adhering tissues, and stored at −20°C for further analysis. Additionally, in order to confirm the success of ovariectomy, atrophied uterus was removed, nicked, and weighed.

### 2.3. Body Composition and Bone Microstructural Properties

Lean mass and fat mass, whole body, and vertebral and tibial BMD and BMC were assessed using dual-energy X-ray absorptiometry (DXA) (Hologic Inc., Bedford, MA, USA) one week prior to removal of the ovaries (baseline) and again two weeks prior to the end of study (final) after rats were anesthetized with ketamine/xylazine (100 mg/5 mg/kg BW). Microarchitectural properties of the right tibia and 4th lumbar vertebra were assessed using a *μ*CT 35 scanner (Scanco Medical, Brüttisellen, Switzerland). Bone and muscle specimens were frozen at −20°C for further examination in a blind manner. Tibia was scanned at the volume of interest (VOI) that was twenty-five slices away from the proximal growth plate in the distal direction (16 *μ*m/slice) to 125 slices. The VOI for lumbar vertebra was twenty-five slices away from the growth plate at each end of the vertebral body and was scanned from the cephalic to the caudal growth plate, resulting in a total of slices. Both bone specimens were scanned at an isotropic voxel resolution of 22 *μ*m3 using a 1024 × 1024 matrix. The integration time per projection was 70 ms and rotational step was 0.36° with a total acquisition time of 150 min per sample. The scanned VOI resulted in a total of 350 and 530 images for tibia and lumbar vertebra, respectively. Morphometric parameters calculated by scanning the VOI included bone volume over total volume (BV/TV), trabecular number (Tb. N), separation (Tb. Sp.), thickness (Tb. Th.), structure model index (SMI), and connectivity density (Conn. D). The obtained 3D microarchitecture allowed visualization of these morphometric measurements.

### 2.4. Blood Parameters

Blood samples were centrifuged (4°C) at 1500*g* for 15 minutes. Serum samples were separated, aliquoted, and stored at −20°C for analyses of 17*β*-estradiol and two of the most widely accepted markers of bone turnover for rats, namely, C-telopeptides of type I collagen (CTX) and bone specific alkaline phosphatase (B-ALP) [19]. These measurements were performed using enzyme linked immunoassay (ELISA) kits from TSZ ELISA (Framingham, MA, USA) and Immunodiagnostic Systems Inc. (Fountain Hills, AZ, USA), respectively, following the manufacturer's protocols.

### 2.5. Statistics

Power calculation: powers for detecting differences were calculated based on a range of possible decreases in BMD of ovx rats when compared to sham-operated rats. The range of possible increases was set beginning at 1% and increased by 1.0% increments to 8%. The power was calculated as follows: probability (rejecting mean (*i*) = mean (*j*) given difference = *x*).

A two-tailed significance level of 0.05 was used. The difference stated in the above probability statement was expressed as a percentage of a reported standard deviation. Thus, 5% increase was one standard deviation, and the square of this value was what was assumed to estimate the pooled variance estimate calculated in the analysis of variance. Six rats per treatment group provided us with a power of more than 0.80 at *α* = 0.05 to detect a difference in BMD of one standard deviation.

*Data Analysis*. For each age group (initial age of 5 months or 10 months), a 3-way randomized design (ovx, ovx + E_2_, and sham) was utilized. SigmaStat 3.5 (Systat Software Inc., San Jose, CA) was used to analyze the data and when a one-way ANOVA indicated any significant differences among the means, Tukey's post hoc test was performed. Statistical significance was set at the *p* < 0.05 level for all analyses. Study was designed to detect the significant cooccurrence of obesity, osteoporosis, and sarcopenia and was terminated upon DXA measurements that provided significant changes in these body tissues. Therefore, the duration of ovarian hormone deficiency was different between the two age groups; three and half months for young animals and five months for older group. To avoid the impact of time differences in duration of ovarian hormone deficiency and estradiol exposure on body composition, the comparison was made among sham and ovx and sham and ovx + E_2_ in each age group only, using one-way ANOVA.

## 3. Results

### 3.1. Food Intake, Physical Activity, and Body and Organ Weights

**Figure 1:**
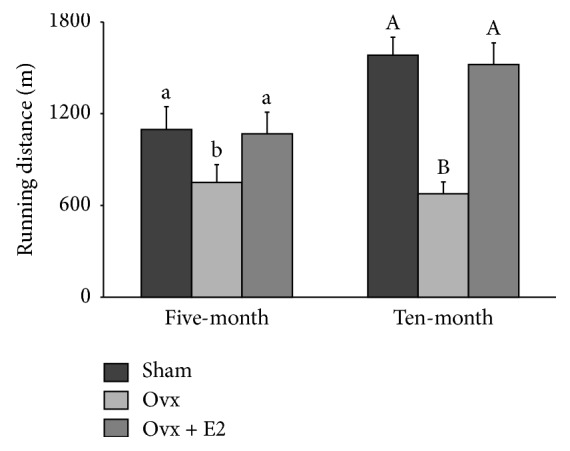
The effects of ovariectomy (ovx) and 17*β*-estradiol (ovx + E_2_) on 24-hour voluntary running distance in rats from the cohorts of initial ages of five months (A) and ten months (B). Values are means ± SEM. Bars that do not share the same superscript letters are significantly (*p* < 0.05) different from each other (*n* = 6 per treatment group). Voluntary running activity measured by activity wheel and distance counter in individual cages for 24 hours between one and two weeks before sacrifice was significantly decreased in older ovx rats (younger ovx rats tended to have a lower activity). E_2_ administration prevented the lower activity level.

Even though ovx rats were pair-fed with the mean food intake of sham animals, the final BWs of ovx rats in both age groups were significantly higher than sham rats (Table 1). E_2_ administration prevented the ovariectomy-induced weight gain. Success of the surgical procedure was evident by atrophy of the uterus that was prevented by E_2_. No significant differences were found between mean heart and liver weights (Table 1). Older ovx rats significantly decreased 24-hour voluntary wheel running activity by 57% compared with sham animals and younger ovx rats tended (*p* = 0.08) to have a lower activity (Figure 1).

### 3.2. Body Composition

The excess BW gain in ovx rats of both age groups was mainly due to a 30% increase of fat mass (*p* = 0.006 and *p* = 0.02, resp., for young and old animals) that was completely prevented by E_2_ (Table 2).

In both age groups ovariectomy significantly reduced whole body BMD, in comparison with sham animals, without affecting BMC due to the simultaneous increases in bone areas (Table 2). Furthermore, the whole body BMD was 9% lower in older animals, reflecting the impact of aging in addition to ovariectomy. E_2_ prevented these alterations in both age groups.

Ovariectomy induced a significant loss of tibial and vertebral BMD in both age groups which was more pronounced in older rats (22% and 16% loss, resp., for tibiae and vertebrae in older animals). The loss of BMC was observed in both tibiae and vertebrae in older ovx rats and only in vertebrae of younger ovx animals. E_2_ prevented the loss of BMD and BMC only in younger animals (Table 3).

### 3.3. Microcomputed Tomography (*μ*CT) Analysis

**Figure 2:**
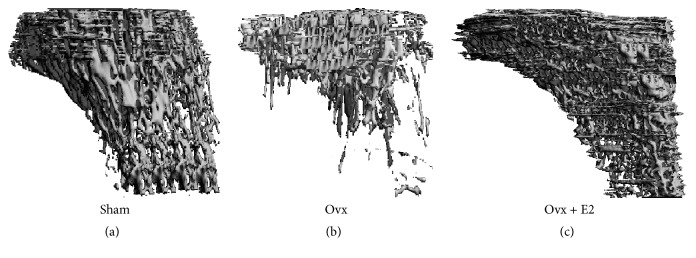
3D trabecular images representative of proximal right tibia of sham (a), ovariectomy (ovx) (b), and 17*β*-estradiol (ovx + E_2_) (c). The images were acquired using *μ*CT (Methods). Ovariectomy decreased trabecular bone structure when compared to sham in rats from the cohort of initial age of ten months. This effect was prevented by E_2_ administration. Similar effects were observed in younger animals (*n* = 6 per treatment group).

**Figure 3:**
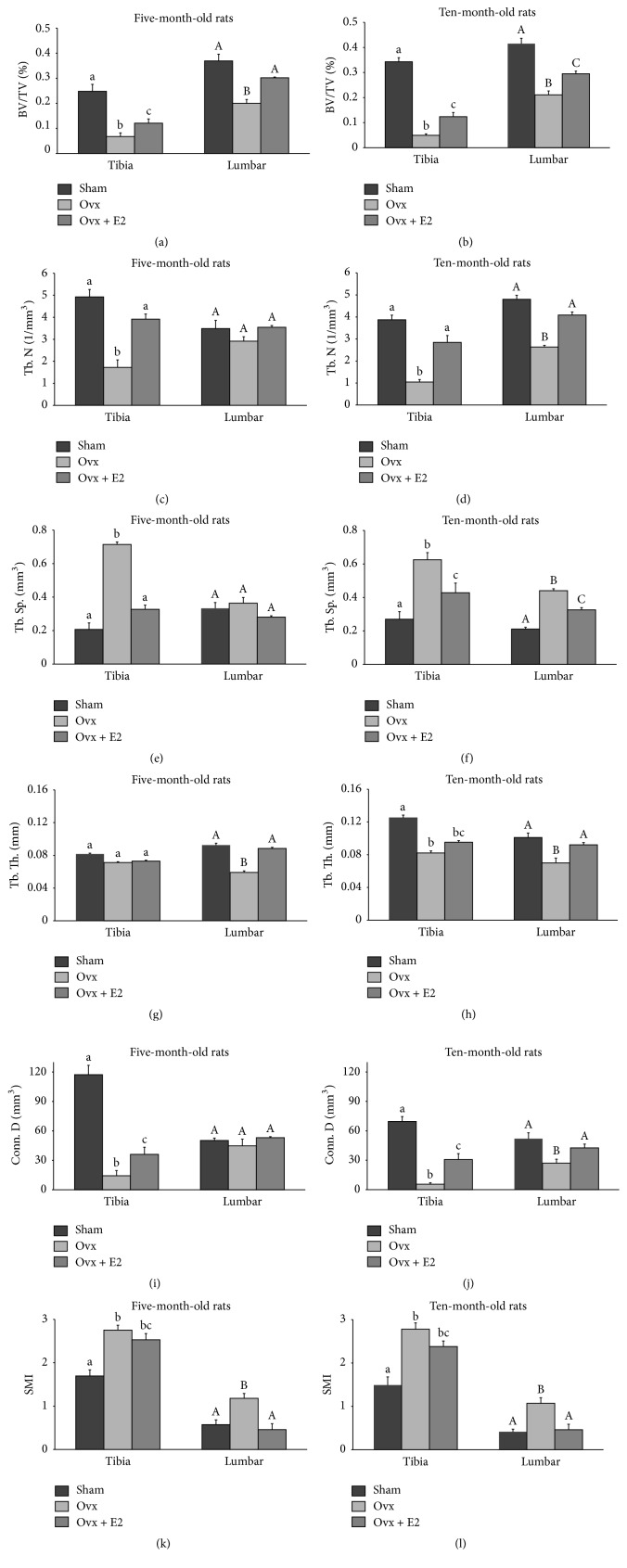
The effects of ovariectomy (ovx) and 17*β*-estradiol (ovx + E_2_) on BV/TV, bone volume/total volume (a and b); Tb. N, trabecular number (c and d); Tb. Sp., trabecular separation (e and f); Tb. Th., trabecular thickness (g and h); Conn. D, connectivity density (i and j); SMI, structure model index (k and l) of right tibiae and 4th lumbar vertebrae in rats from the cohorts of initial ages of five (a, c, e, g, i, and k) and ten months (b, d, f, h, j, and l). Values are means ± SEM. Bars that do not share the same superscript letters are significantly (*p* < 0.05) different from each other. Small and large letters represent tibia and lumbar, respectively (*n* = 6 per treatment group). Ovariectomy significantly decreased tibia and lumbar trabecular BV/TV, bone volume/total volume, in both age groups that was only prevented by estrogen, in the vertebral bone of younger animals (a and b). In both age groups the mean proximal tibial values for Tb. N were significantly reduced following ovariectomy that was prevented by E_2_. However, a significant loss of vertebral Tb. N only was observed in ovx rats (c and d). Tb. Sp. significantly increased in both tibiae and vertebrae of the older ovx rats and tibiae of the younger ovx group; only the latter was prevented by E_2_ (e and f). Reduced levels of Tb. Th. reached a significant level only in the tibiae of the older ovx animals which was not prevented by E_2_ (g and h). Conn. D was reduced in tibiae of both ovx age groups and vertebrae of the older animals. The latter was prevented by estrogen (i and j). SMI values for both bones increased in both ovx age groups which was prevented by E_2_ only in vertebral bones (l).

Representative images of the 3D trabecular microstructures of proximal tibiae of the three groups in the older (initial age of 10 months) cohort are presented in Figure 2. Ovariectomy significantly decreased trabecular BV/TV in tibiae (73% and 86%, resp., in young and old rats) and lumbar (46% and 49%, resp., for young and old rats), compared with their corresponding sham animals (Figures 3(a) and 3(b)). E_2_ only prevented the vertebral trabecular bone loss in younger animals.

The mean proximal tibial values for Tb. N were significantly reduced in both age groups (65% and 73%, resp., in young and old rats) that were prevented by E_2_. Only older ovx rats experienced a significant loss (45%) of vertebral Tb. N (Figures 3(c) and 3(d)).

Tb. Sp. significantly increased in both tibiae and vertebrae of the older ovx rats (74% and 46%, resp.) and tibiae of the younger ovx group; only the latter was prevented by E_2_ (Figures 3(e) and 3(f)).

Both ovx age groups experienced a reduced Tb. Th. that reached a significant level only in the tibiae of the older ovx animals which was not prevented by E_2_ (Figures 3(g) and 3(h)).

A significant reduction of Conn. D was observed in tibiae of both ovx age groups and vertebrae of the older animals. The latter was prevented by E_2_ (Figures 3(i) and 3(j)).

SMI, which quantifies the pattern of trabeculae as either more rod- or plate-like, was 1.69 and 0.572 in tibiae and vertebrae of younger sham rats, respectively (Figure 3(k)). SMI values for both bones increased in ovx rats; that is, SMI = 2.753 and 1.18 in tibiae and vertebrae, respectively, indicating a shift to less favorable rod-like trabecular bone. Similarly in older animals SMI values for tibiae and vertebrae were both increased compared to those of sham values, that is, 2.78 versus 1.49 and 1.07 versus 0.41 (Figure 3(l)). The higher SMI values in ovx rats indicated that ovariectomy made the remaining bone inferior. E_2_ prevented the vertebral increase in SMI (Figure 3(l)).

### 3.4. Muscle Mass and Ratios

Muscle mass was not significantly affected by ovariectomy. However, when SOL and GAS values were normalized to BW, mean GAS value in the younger ovx + E_2_ group tended (*p* = 0.09) to increase compared with that of sham group (Table 4).

In older ovx rats, normalized SOL value tended (*p* = 0.094) to decrease and GAS value was significantly lower than that of the sham and/or ovx + E_2_ groups. E_2_ prevented the loss of SOL and GAS by 50% and 100%, respectively. In both age groups the ratio of tibial BMC to combined GAS and SOL values of ovx group was significantly lower (12% and 21%, resp., for young and old animals) than that of sham and/or ovx + E_2_ groups which was prevented by estrogen (Table 4).

### 3.5. Blood Parameters

Ovariectomy significantly lowered serum E_2_ levels and increased B-ALP (28% and 26% resp., for ovx young and old rats) and CTX (31% and 34% resp., for ovx young and old animals) in both age groups. Although E_2_ administration in both age groups lowered B-ALP and CTX levels, they were higher than that of sham animals (Table 5).

## 4. Discussion

Our data suggest that ovarian hormone deficiency underlies pathophysiologic obesity followed by osteoporosis that also extended to sarcopenia. Since pair-feeding the animals ruled out the confounding factor of energy intake, excess fat mass suggests a significant decrease in energy expenditure in both age groups. This was partially reflected by decreased 24-hour voluntary wheel running activity that was prevented by E_2_. Estradiol administration prevented both ovariectomy-induced increase in fat mass and decrease in lean mass including bone mass. Therefore, these alterations in body composition and in physical activity can be attributed to estradiol, the main ovarian hormone. Advancing age in these younger age ovarian deficient animal models negatively affects body composition, years before traditional diagnosis of these age-related diseases. We also observed a site specific, age independent protective effect of E_2_ on muscle mass. Moreover, our data suggest an anticatabolic role for E_2_ in younger rats, reflected by higher ratio of normalized mean GAS value in the ovx + E_2_ group compared with that of corresponding mean sham value.

Ovarian hormones may have a direct role on adiposity by increasing hormone-sensitive lipase activity, lipolytic effects of epinephrine, and beta oxidation of fatty acids [6–8]. In addition to the direct effect of ovarian hormone on adipose tissue, it may indirectly affect adiposity via regulation of food intake and energy expenditure through the hypothalamus, the key regulator of food intake and energy homeostasis in the brain [5, 9]. In the present study, BW was greater for ovx animals in both age groups despite similar food consumption compared with those of sham-operated rats. The observed increase in BW was associated with increased fat mass suggesting a shift in energy metabolism. Reduced voluntary physical activity may, in part, explain these unfavorable ovariectomy-induced body compositional changes. Similar results have been reported from ovx animals, displaying an increase in food consumption and a decrease in voluntary physical activity [20, 21]. Consistent with animal findings, human studies have also demonstrated that women, particularly a few years after the onset of menopause, gain weight because of increased fat mass and that postmenopausal women have greater appetite with less desire for physical activity [22].

In addition to the interrelationship between E_2_ and adiposity, it is known to be positively associated with bone mass. Despite the commonly held belief that obesity is beneficial to maintain bone health [23], recent findings show an inverse relationship between fat mass and bone mass in both humans and laboratory animals [3, 4]. In the present study, our obese ovx rats lost bone mass and quality. We observed a significant loss of BMD and BMC in whole body and regional bones in the ovx groups, and this loss was more pronounced in our older ovx animals. The microarchitectural properties of the trabecular bone in both tibiae and lumbar vertebrae, in contrast to the effects of ovariectomy and ovx + E_2_ with those of sham values in each age group, clearly demonstrate the importance of ovarian hormones. Our results also confirmed that ovariectomy caused loss of tibial BV/TV, Tb. N, and Conn. D and increased Tb. Sp. although the degree of loss was greater in older rats than in younger animals. These findings indicate that advancing age, aside from other factors, such as ovarian hormone deficiency that deleteriously affect bone microstructure, can be an independent factor affecting bone loss. This could be related in part to age-dependent changes in bone mesenchymal stem cells [24] and the role of estrogen in directing their differentiation into either adipocytes or osteoblasts [25]. Our results are consistent with findings of other investigators reporting that in addition to BMD bone microarchitecture deteriorates because of ovarian hormone deficiency in both women and in ovx animals [26, 27].

Unlike the observations made in postmenopausal women and osteoporotic men [28] who often experience increases in trabecular thickness as a result of reduced trabeculae, our results showed a decrease in Tb. Th. following ovariectomy. This, in part, can be explained by the morphological differences between the two species in bone and in muscle structures. Because of their structural physiology, rats do not experience fractures even when severe bone loss is evident [29]. Alternatively, the skeletal system in humans compensates for the loss of trabeculae through an increase in the thickness of the remaining trabeculae [30]. Although this may postpone fracture, continued bone loss in humans eventually leads to fracture because of increased stress associated with thinning and perforation of trabeculae [30]. In the present study, *μ*CT analysis indicated that E_2_ treatment partially prevented the loss of trabecular structures, findings which concur with observations by Lark et al. [31, 32]. Administration of E_2_ also had a more pronounced modulating effect in younger rats than in older rats.

In addition to the structural parameters, other morphological parameters, such as SMI, reflect bone quality and strength. The SMI value is based on surface density and thickness of a 3-dimensional structure. Its value is multiplied by a factor to yield more practical numbers, 0 for ideal plates, 3 for ideal rods, and 4 for true spheres [33]. A plate-like structure is generally stronger than a rod-like structure [33]. The SMI values in both age categories were higher in ovx rats compared to corresponding sham animals, indicating that ovarian hormone deficiency not only causes bone loss, but also, at the same time, decreases the quality of the remaining bone.

In humans and rodents, both estrogen receptors (ERs), ER*α* and ER*β*, are expressed in skeletal muscle, making it a target tissue of estrogen action [10]. Although a number of studies support a link between muscle mass and estrogen [34, 35], not all results from human and animal studies are in agreement about the relationship between estrogen and skeletal muscle. Results of human studies are inconsistent because of the variety of study designs (e.g., cross-sectional versus longitudinal) and because of age and gender differences of the samples examined [34]. Other contributing factors adding to these inconsistencies among animal studies include variations in animal models used, the duration of experiments, and the age of the animals [13, 14]. In the present study, we were able to demonstrate the coexistence of obesity with sarcopenia in the older osteopenic ovx rats. Our findings are consistent with several reports by other investigators [13, 14] which have reported that ovarian hormone deficiency delays recovery of the skeletal muscle mass from disuse-induced muscle atrophy in rats. ER*α* interacts with nuclear troponin I in fast skeletal muscle [36]. Interestingly, nuclear localization of troponin I has also been identified in cardiac muscle [37] and could be associated with some forms of cardiomyopathy that alter myocyte size [38]. In addition, nuclear localization of troponin T, which is a binding partner of troponin I in striated muscles, has been shown to vary with age, and this may be associated with some aspects of sarcopenia [39–41]. Interestingly, we observed a greater response to E_2_ manipulation in GAS skeletal muscle compared with SOL that may in part be explained by higher proportion of ER*α* on fast-twitch muscle fibers. Also, in cross-sectional studies, a positive relationship between muscle mass and plasma levels of estrogen, estrone, and estradiol in women has been reported suggesting a faster decline of muscle mass in premenopausal women [35]. Furthermore, consistent with Frost's hypothesis and data from postmenopausal women [42, 43], we observed a significant decline in the ratio of tibial BMC to skeletal muscle mass in our ovx rats in both age groups, an effect that was prevented by E_2_. These findings suggest that E_2_ may lower the required load to prevent bone loss or promote bone formation.

Consistent with available literature we observed a site specific protective effect of E_2_ on skeletal muscle [43]. Our estrogen administered rats maintained muscle weights, irrespective of age. Furthermore, our findings revealed an apparent anticatabolic effect of E_2_ in five-month-old animals, as the mean normalized GAS and SOL weights of E_2_ treated rats were higher than both sham and ovx groups. The potential anticatabolic effect of E_2_ on skeletal muscle has been reported using young ovx rats and human studies with younger subjects; however, there may be less of an effect of E_2_ with advancing age, but the strength of evidence to support this contention is minimal.

Ovx rat is the most commonly used model demonstrating bone loss [19]. Similar to menopause, the ovx rats experience increased rates of bone resorption and bone formation with the rate of resorption exceeding that of formation [19] which results in a net loss of bone mass. In our study, the bone resorption marker CTX was significantly elevated in both obese ovx rats and exceeded that of bone formation marker B-ALP in these animals compared to corresponding sham rats of both age groups. E_2_ deficiency in the ovx rat model appears to be the most appropriate method available for modeling excess resorption not accompanied by formation deficit [19]. In addition to loss of bone mass, ovx rats increase fat mass along with a gradual decline in muscle mass. Therefore, the ovx rat could be used as an experimental model to conduct obesity translational research without the confounding effects of diet or genetic background.

Our findings suggested that the cooccurrence of obesity and sarcopenia along with osteoporosis may be found in ovarian hormone deficient women as a result of several factors which work together and exacerbate mobility disability. First, the gradual reduction in female sex hormones by advancing age can affect energy intake and expenditure and potentially contribute to added fat accumulation and obesity. Low levels of ovarian hormones are linked to decreased spontaneous physical activity and to decreased energy expenditure, leading to an increase in fat mass even without additional energy intake. Second, excess adiposity may impair bone and muscle mass and function on a physiological basis through certain mechanisms affected by systemic inflammation and impaired metabolic function. Third, the excess weight, along with bone and muscle loss, may result in an impaired functionality that accounts for a higher rate of obesity and obesity related medical complications. All of these factors may contribute to frailty and to increased risk of mortality in women with low levels of ovarian hormones.

## 5. Conclusion

In summary, using five- and ten-month-old ovx rats (initial age of five or ten months) enabled us to disentangle the effect of ovarian hormone deficiency from the complex multifactorial process of chronological aging in the female body while controlling for energy intake and measuring physical inactivity. Our findings in these two age cohorts ovx SD rats suggested that removal of ovaries alters BC similar to what can be manifested as osteosarcopenic obesity in postmenopausal women. While previous studies have investigated these age-related disease conditions in isolation, in older animals that mimic the same age as of menopausal women, to the best of our knowledge, the cooccurrence of all of these three conditions has not yet been examined. To understand the mechanistic relationship between these diseases, our study demonstrates the utility of ovx rat as a proper model for future translational research. Future studies are needed to examine the effects of ovarian hormone deficiency on changes that occur in energy metabolism in fat and fat-free mass and the roles that E_2_ plays in promoting homeostasis and the mechanistic crossroads that lead to divergent outcomes following E_2_ exposure. Also, the related clinical dimensions and interactions of obesity, sarcopenia, and osteoporosis need to be investigated.

## Figures and Tables

**Table 1:** The effects of ovariectomy (ovx) and 17*β*-estradiol (ovx + E_2_) on food intake, body, and organ weight in rats with initial ages of five and ten months. Despite pair-feeding the animals, ovariectomy significantly increased body weight and caused atrophy of uterine tissue without any effects on heart or liver weights.

Parameter	5-month-old			10-month-old		
	Sham	Ovx	Ovx + E_2_	Sham	Ovx	Ovx + E_2_
Food intake (g/day)	16.38 ± 9.1	16.39 ± 9.8	16.05 ± 9.4	15.65 ± 9.1	15.68 ± 9.8	15.01 ± 9.4
Body weight (g)						
Initial	294.00 ± 6.96	293.33 ± 6.65	293.67 ± 4.99	369.40 ± 6.96	370.00 ± 6.65	361.98 ± 4.99
Final	366.40 ± 9.8^a^	400.40 ± 9.8^b^	350.33 ± 13.38^a^	421.80 ± 9.8^A^	474.00 ± 10.35^B^	404.00 ± 6.91^A^
Organ weight (mg)						
Uterus	0.618 ± 0.05^a^	0.098 ± 0.01^b^	0.287 ± 0.03^c^	0.733 ± 0.05^A^	0.140 ± 0.02^B^	0.232 ± 0.01^C^
Liver	10.640 ± 0.653	9.362 ± 0.236	8.750 ± 0.617	12.98 ± 1.33	13.22 ± 2.03	10.66 ± 0.06
Heart	1.050 ± 0.057	0.965 ± 0.0293	0.950 ± 0.029	1.186 ± 0.029	1.167 ± 0.035	1.125 ± 0.027

Values are means ± SEM. Values that do not share the same superscript letters in each age category are significantly (*p* < 0.05) different from each other (*n* = 6 per treatment group).

**Table 2:** The effects of ovariectomy (ovx) and 17*β*-estradiol (ovx + E_2_) on body composition in rats with initial ages of five and ten months. Ovariectomy induced a significant increase in total fat mass and a decrease in bone mineral density in both age groups and a decrease in lean mass in the older group. E_2_ administration prevented ovx-induced alterations in body composition.

Parameters	5-month-old			10-month-old		
	Sham	Ovx	Ovx + E_2_	Sham	Ovx	Ovx + E_2_
Total fat mass (g/cm^2^)						
Baseline	69.4 ± 6.5	70.5 ± 5.9	73.2 ± 5.5	102.6 ± 14.9	100.3 ± 12.8	101.3 ± 10.0
Final	172.0 ± 10.5^a^	226.0 ± 8.7^b^	170.2 ± 13.2^a^	218.6 ± 11.5^A^	284.0 ± 7.5^B^	217.7 ± 7.7^A^
Total lean mass (g)						
Baseline	204.6 ± 10.6	203.8 ± 7.5	203.0 ± 7.1	221.4 ± 9.3	231.2 ± 8.3	219.8 ± 6.6
Final	167.0 ± 11.0^a^	146.2 ± 9.1^b^	160.2 ± 9.6^a^	168.2 ± 10.5	152.2 ± 11.1	160.2 ± 4.6
Total BMD (g/cm^2^)						
Baseline	0.159 ± 0.0008	0.160 ± 0.004	0.161 ± 0.002	0.186 ± 0.007	0.187 ± 0.003	0.181 ± 0.003
Final	0.173 ± 0.003^a^	0.158 ± 11.96^b^	0.172 ± 0.004^a^	0.181 ± 0.008^A^	0.169 ± 0.002^B^	0.179 ± 0.006^A^
Total BMC (g)						
Baseline	8.940 ± 0.108	8.800 ± 0.144	9.100 ± 0.224	11.460 ± 0.93	11.350 ± 0.19	10.517 ± 0.53
Final	11.54 ± 0.42	11.60 ± 0.004	11.18 ± 0.004	13.60 ± 0.80	13.73 ± 0.34	13.17 ± 0.38

Values are means ± SEM. Values that do not share the same superscript letters in each age category are significantly (*p* < 0.05) different from each other. BMD, bone mineral density; BMC, bone mineral content (*n* = 6 per treatment group).

**Table 3:** The effects of ovariectomy (ovx) and 17*β*-estradiol (ovx + E_2_) on bone mineral density and bone mineral content of the 4th lumbar and right tibia in rats with initial ages of five and ten months. The mean tibial and vertebral bone mineral density and bone mineral content of ovx rats were significantly lower in comparison with sham rats in both age groups and E_2_ administration prevented the bone loss.

Parameters	5-month-old			10-month-old		
	Sham	Ovx	Ovx + E_2_	Sham	Ovx	Ovx + E_2_
4th lumbar						
BMD (g/cm^2^)	0.246 ± 0.005^a^	0.195 ± 0.002^b^	0.240 ± 0.005^a^	0.266 ± 0.016^A^	0.207 ± 0.004^B^	0.230 ± 0.004^C^
BMC (g)	0.130 ± 0.007^a^	0.089 ± 0.005^b^	0.128 ± 0.006^a^	0.162 ± 0.017^A^	0.115 ± 0.003^B^	0.127 ± 0.005^C^
Right tibia						
BMD (g/cm^2^)	0.211 ± 0.003^a^	0.183 ± 0.004^b^	0.209 ± 0.002^a^	0.240 ± 0.009^A^	0.204 ± 0.004^B^	0.218 ± 0.004^C^
BMC (g)	0.314 ± 0.003	0.292 ± 0.007	0.309 ± 0.007	0.375 ± 0.002^A^	0.331 ± 0.009^B^	0.338 ± 0.006^C^

Values are means ± SEM. Values that do not share the same superscript letters in each age category are significantly (*p* < 0.05) different from each other. BMD, bone mineral density; BMC, bone mineral content (*n* = 6 per treatment group).

**Table 4:** The effects of ovariectomy (ovx) and 17*β*-estradiol (ovx + E_2_) on muscle mass and normalized muscle mass in rats with initial ages of five and ten months. Ovariectomy did not affect the muscle mass but decreased ratio of tibial bone mineral content to combined muscle mass in both age groups and lowered normalized values of gastrocnemius muscle to body weight in older rats that were prevented by E_2_ administration.

Parameters	5-month-old			10-month-old		
	Sham	Ovx	Ovx + E_2_	Sham	Ovx	Ovx + E_2_
Muscle mass						
Gastrocnemius (g)	1.7814 ± 5.886	2.0763 ± 4.442	1.8828 ± 5.747	2.228 ± 1.09	2.242 ± 0.004	2.129 ± 0.054
Soleus (mg)	135 ± 9.2	163 ± 9.1	139 ± 12	150 ± 7.0	151 ± 9.1	140 ± 5.1
MM/BW (mg/g)						
Gastrocnemius	4.881 ± 0.0231	5.191 ± 0.0118	5.458 ± 0.0197	5.28 ± 0.005^A^	4.73 ± 0.011^B^	5.28 ± 0.015^A^
Soleus	0.401 ± 0.029	0.408 ± 0.024	0.402 ± 0.034	0.37 ± 0.001	0.31 ± 0.02	0.34 ± 0.003
BMC/CMM (*μ*g/mg)	16.483 ± 0.596^a^	13.042 ± 0.125^b^	14.872 ± 0.400^a^	15.728 ± 0.795^A^	13.879 ± 0.304^B^	14.541 ± 0.439^A^

Values are means ± SEM. Values that do not share the same superscript letters in each age category are significantly (*p* < 0.05) different from each other. MM, muscle mass; BW, body weight, BMC, right tibia bone mineral content; CMM, combined muscle mass (*n* = 6 per treatment group).

**Table 5:** The effects of ovariectomy (ovx) and 17*β*-estradiol (ovx + E_2_) on blood parameters in rats with initial ages of five and ten months. Ovariectomy significantly lowered serum E_2_ and increased B-ALP and CTX in both age groups.

Parameter	5-month-old			10-month-old		
	Sham	Ovx	Ovx + E_2_	Sham	Ovx	Ovx + E_2_
E_2_ (ng/mL)	18.50 ± 4.5^a^	7.89 ± 3.8^b^	24.19 ± 3.1^c^	21.07 ± 7.1^A^	8.45 ± 2.1^B^	28.10 ± 4.5^C^
B -ALP (U/L)	24.16 ± 13.51^a^	29.77 ± 8.81^b^	25.10 ± 11.43^a^	20.56 ± 7.75^A^	27.35 ± 9.02^B^	24.01 ± 8.8^C^
CTX (ng/ml)	84.25 ± 9.05^a^	110.22 ± 11.01^b^	90.03 ± 10.03^c^	77.50 ± 9.05^A^	103.95 ± 11.02^B^	82.32 ± 8.01^C^

Values are means ± SEM. Values that do not share the same superscript letters in each age category are significantly (*p* < 0.05) different from each other. CTX, C-telopeptides of type I collagen; B-ALP, bone specific alkaline phosphatase; and E_2_, estrogen (*n* = 6 per treatment group).
